# Mr. Goodall's Case of Hernia

**Published:** 1805-11-01

**Authors:** J. Goodall

**Affiliations:** Epworth, Lincolnshire


### *To the Editors of the Medical and Physical Journal.*

GENTLEMEN.

AS there is no complaint more distressing to the patient, nor more perplexing to the practitioner, than every species of Hernia, I have taken the liberty to send you the following case, which, if you deem of sufficient importance to to merit a place in your much esteemed Journal, I beg you will insert it.

On the evening of 29th August last, I was sent for to Mr. I. G. 73 years of age; he has been a very healthy man, but has had a rupture upwards of seventeen or eighteen years, for which he never wore a truss. Upon enquiry I found a very large portion of the bowel had descended into the scrotum, on the left side, which formed a tumor nearly as large as an infant's head; a most acute pain is felt in the tumor, attended with uneasiness over the whole abdomen; bowels costive, sickness at stomach; had vomited several times, but little thirst; pulse rather full, and 86 strokes in a minute.

I have met with several very distressing cases of rupture, and have always, hitherto, been exceedingly foiled in my intentions towards relief; glysters, castor oil, and the different neutral salts, sometimes bleeding, with small doses of opium, have been of very little service after many hours, and even days, regular perseverance in the use of them; but fortunately for my patient, the subject of these observations, a very interesting case of the same kind, cured by opiates, and related by Dr. Ross, of Montrose, fell into my hands; I was therefore determined to try what effect a strong opiate would have in the present instance.

Without paying the least attention to the costive state of his bowels, I immediately gave him a mixture composed of 150 drops of laudanum, ℥ijß cinnamon water, and ʒß syr. white poppies; one-third to be taken immediately, and to be repeated at intervals of an hour. The first dose brought on considerable relaxation of the part, and he obtained relief in two hours, fell asleep, and did not wake till four o'clock in the morning. He then had some little return of pain and tension of the belly, took a second dose of the mixture, fell asleep again, and at seven o'clock this morning (August 30) the bowel had returned into its natural situation. He expressed great pleasure, by saying he was as well as ever he was in his life.

It may be necessary to observe, that the laudanum did not occasion the least sickness at stomach. He took two doses of Castor oil, waiting two days betwixt each dose; since that time he has remained perfectly free from the complaint. I shall not make any comments; I merely wished to simply state the case, for the information of your readers, and the good of the afflicted in general.

I am, &c.

*Epworth, Lincolnshire, Sept.* 18, 1805. J. GOODALL.

